# Supplementary material for: An active mechanical Willis meta-layer with asymmetric polarizabilities
Source: Nat Commun. 2020 Jul 23;11:3681. doi: 10.1038/s41467-020-17529-2 (PMC7378557; doi:10.1038/s41467-020-17529-2)
Supplement: Supplementary file 1 — Supplementary Information [file 41467_2020_17529_MOESM1_ESM.pdf]

SUPPLEMENTARY INFORMATION

An active mechanical Willis meta-layer with asymmetric polarizabilities

Chen et al.

Supplementary Note 1: Description of Transfer Function *G*

In experiments, a system identification technique [1] is implemented to identify the linear invariant transfer function, *G*, which is defined by $G = \frac{\bar{V}_s}{\bar{V}_{a1}} = \frac{\bar{V}_s}{\bar{V}_{a2}}$ with $\bar{V}_{a1}$ and $\bar{V}_{a2}$ being trail actuating voltages, and $\bar{V}_s$ being the corresponding sensing voltage induced by the trail actuating voltage. Therefore, the feedback components in the sensing signal are eliminated, and the control system becomes feedforward (see Fig. 2a). To experimentally realize *G*, a six-order pole-zero transfer function is firstly predefined, whose parameters are then determined through time series signal analyses, such that *G* can precisely mimic mechanical feedbacks.

Supplementary Note 2: Feedforward Control Loop

The schematic of the feedforward control loop is shown in **Supplementary Figure 1**. As illustrated in the figure, the piezoelectric sensor is connected to a charge amplifier and a low-pass filter (the detailed circuit designs are given in subset figures). Subsequently, the output signal from this low-pass filter feeds to a digital control unit (dSPACE SCALEXIO 6001) implemented with a band-pass filter and three transfer functions H_1, H_2 and *G* connected by two subtract loops. The two output signals from the digital controller are applied to the two actuators, respectively, through two low-pass filters and two voltage amplifiers. The detailed parameters of those analog circuits are given in **Supplementary Table 1.** Circuit parameters of the fabricated control circuit system

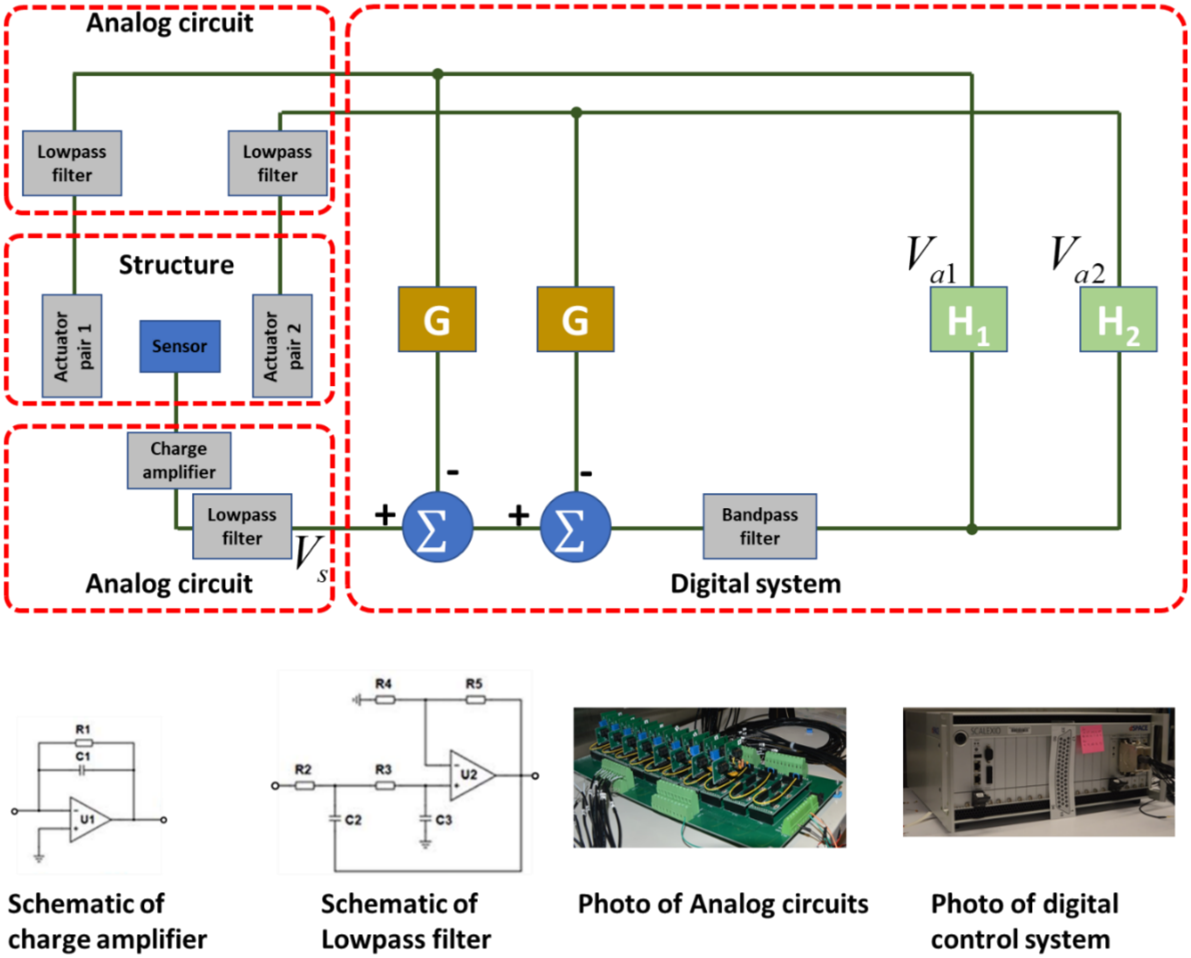

Supplementary Figure 1. Diagram of the feedforward control loop.

Supplementary Table 1. Parameters of analog circuits.

**Geometric parameters (mm)**					
R1	1MΩ	R2,R3	3 kΩ	R4, R5	1 kΩ
**C1**	100 pF	C2, C3	2 nF		
Op-amp	OPA445	Controller	dSPACE SCALEXIO 6001		

Supplementary Note 3: Derivation of transfer functions H_1 and H_2

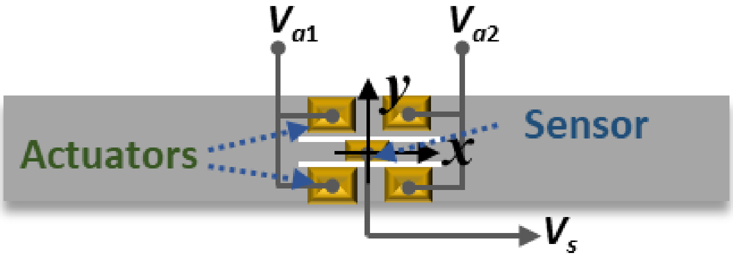

Supplementary Figure 2. Schematic of a unit cell of the Willis meta-layer.

To derive transfer functions H_1 and H_2 achieving desired wave transmission and reflection properties, we consider a 1D setting with a single meta-layer embedded into a host beam as shown in **Supplementary Figure 2**. Assume an incident wave coming from the left with the displacement

$$w_{in} = w_0 e^{ikx}. \tag{1}$$

Applying a voltage, V_{a1}, on the left pair of piezoelectric patches generates two waves propagating to the left and right symmetrically, where the displacement wave field can be expressed

$$\begin{aligned} w_{a1} &= \kappa_a V_{a1} e^{ik(x+a)}, x > -a, \\ w_{a1} &= \kappa_a V_{a1} e^{-ik(x+a)}, x < -a, \end{aligned} \tag{2}$$

where κ_a is the effective electromechanical coupling coefficient of the actuating piezoelectric patches, and a denotes the distance of the left pair of piezoelectric patches from the origin. Similarly, applying another voltage, V_{a2}, on the right pair of piezoelectric patches generates two waves with the displacement wave field being

$$\begin{aligned} w_{a2} &= \kappa_a V_{a2} e^{ik(x-a)}, x > a, \\ w_{a2} &= \kappa_a V_{a2} e^{-ik(x-a)}, x < a. \end{aligned} \tag{3}$$

Consequently, the total displacement wave field reads

$$\begin{aligned} w_{t_l} &= (w_0 + \kappa_a V_{a1} e^{ika} + \kappa_a V_{a2} e^{-ika}) e^{ikx}, \quad x > a, \\ w_{r_l} &= (\kappa_a V_{a1} e^{-ika} + \kappa_a V_{a2} e^{ika}) e^{-ikx}, \quad x < -a. \end{aligned} \tag{4}$$

The transmission and reflection coefficients can be directly obtained as

$$\begin{aligned} T_l &= \frac{\kappa_a V_{a1} e^{ika} + \kappa_a V_{a2} e^{-ika}}{w_0} + 1, \\ R_l &= \frac{\kappa_a V_{a1} e^{-ika} + \kappa_a V_{a2} e^{ika}}{w_0}. \end{aligned} \tag{5}$$

Equation (5) indicates that both the transmitted and reflected wave fields can be independently controlled by properly applying voltages on the two actuating pairs in the Willis meta-layer.

On the other hand, to obtain desired transmission and reflection coefficients, the voltages applied on the actuators should read

$$\begin{aligned} V_{a1} &= \frac{(T_l - 1) w_0 e^{ika} - w_0 R_l e^{-ika}}{\kappa_a e^{2ika} - \kappa_a e^{-2ika}}, \\ V_{a2} &= \frac{(T_l - 1) w_0 e^{-ika} - w_0 R_l e^{ika}}{\kappa_a e^{-2ika} - \kappa_a e^{2ika}}. \end{aligned} \tag{6}$$

As demonstrated in design principles, the sensing voltage of the piezoelectric sensor feeding to transfer functions H_1 and H_2 only contains the incident wave component as

$$\bar{V}_s = \kappa_s w_0, \tag{7}$$

where κ_s denotes the other electromechanical coupling coefficient of the sensor.

Therefore, combining Eqs. (6) and (7), transfer functions H_1 and H_2 for simultaneous control of transmitted and reflected waves can be determined

$$H_1 = \frac{V_{a1}}{\bar{V}_s} = \frac{(T_l - 1)e^{ika} - R_l e^{-ika}}{\kappa_s \kappa_a e^{2ika} - \kappa_s \kappa_a e^{-2ika}},$$
$$H_2 = \frac{V_{a2}}{\bar{V}_s} = \frac{(T_l - 1)e^{-ika} - R_l e^{ika}}{\kappa_s \kappa_a e^{-2ika} - \kappa_s \kappa_a e^{2ika}}. \tag{8}$$

Note that frequency responses of transfer functions, H_1 and H_2, given in Eq. (8), are derived from frequency-domain analyses, such that causality may not be guaranteed in time domain. In the experiments, we made use of two sixth-order low-pass infinite impulse response (IIR) filters to construct the two transfer functions. To achieve the frequency responses described in Eq. (8) at each of the single frequencies, we finely tuned the amplification ratio of the filters and added proper time delay on the output signals during each of the reconfiguration steps. It can be clearly seen from experiments that the linear signal processing filters in the time domain are inevitably causal (see Source Data for the coefficients of the filter).

Furthermore, changing the incident direction from the left to the right, the total displacement wave field becomes

$$w_{r_r} = (\kappa_a V_{a1} e^{ika} + \kappa_a V_{a2} e^{-ika}) e^{ikx}, \qquad x > a,$$
$$w_{t_r} = (w_0 + \kappa_a V_{a1} e^{-ika} + \kappa_a V_{a2} e^{ika}) e^{-ikx}, \qquad x < -a. \tag{9}$$

Comparing Eqs. (4) and (9), transmission and reflection coefficients for the incidence from the right, T_r and R_r, are different from those with the incidence from the left side with the transfer function given in Eq. (8) and their relations can be express as

$$T_r = 1 + R_l,$$
$$T_l = 1 + R_r. \tag{10}$$

Therefore, the Willis meta-layer is intrinsically nonreciprocal.

Supplementary Note 4: Transmittance of the meta-layer without electrical control

We calculate transmittance of the meta-layer without electrical control at frequencies of interested using numerical simulations (**Supplementary Figure 3**). It is found transmittance is very close to 1 in the presence of the passive meta-layer. Therefore, scattering in the passive state is negligible.

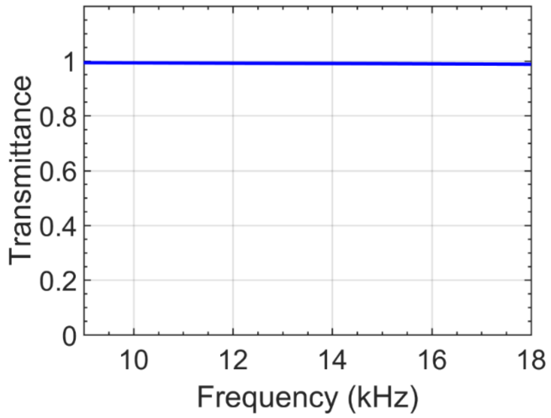

Supplementary Figure 3. Numerically calculated transmittance of the meta-layer without electrical control at frequencies from 9 to 18 kHz.

Sample fabrication and experimental setup of a beam with Willis meta-layer

Supplementary Figure 4 shows the fabricated Willis meta-layer in a beam as well as experimental setup for wave field measurements. To fabricate the sample, two slots were firstly cut out of from a stainless steel strip with fiber laser machine to form sensing and actuating beams. A piezoelectric patch (APC 850) was bonded at the center of the sensing beam. Two pairs of piezoelectric patches (APC 850) was then symmetrically attached on the actuating beams. A conductive epoxy (Chemtronics) is used for those bonding. Two additional piezoelectric patches were attached on the left and right sides of the meta-layer to generate waves propagating from different directions.

Geometric and material parameters of the Willis meta-layer and the host beam are listed in **Supplementary Table 2**. A Polytec PSV-400 3D scanning Laser Doppler Vibrometer (3D SLDV) system was used to measure the velocity fields on the host beam. In experiments, 10-peak tone-burst signals were generated in a built-in function generator in the 3D SLDV system. The signals were then amplified through a power amplifier, which were finally applied on the left/right piezoelectric patches to generate incident waves.

Supplementary Table 2. Geometric and material parameters of the proposed beam with Willis meta-layer

**Geometric parameters (mm)**					
$\boldsymbol{L}$	2000	L_{st}	12	w_s	3.5
$\boldsymbol{L}_1$	300	L_{p1}	3.0	h	3
$\boldsymbol{L}_2$	300	L_{p2}	2.9	h_{p1}	1.0
$\boldsymbol{L}_3$	400	w	21.9	h_{p2}	0.6
$\boldsymbol{L}_4$	400	w_{st}	0.8	a	4.3
$\boldsymbol{L}_5$	300	w_a	8.0		
**Material properties (Steel)**					
$\mathbf{E_b}$	210.0 GPa	G_b	80.8 GPa	ρ_b	7800.0 kg m^{-3}
**Material properties (APC 850)**					
$\boldsymbol{s_{11}^E}$	16.4×10^{-12} m^2 N^{-1}		d_{33}	374.0×10^{-12} C N^{-1}	
$\boldsymbol{s_{33}^E}$	18.8×10^{-12} m^2 N^{-1}		d_{31}	-171.0×10^{-12} C N^{-1}	
$\boldsymbol{s_{44}^E}$	47.5×10^{-12} m^2 N^{-1}		d_{15}	584.0×10^{-12} C N^{-1}	
$\boldsymbol{s_{12}^E}$	-5.74×10^{-12} m^2 N^{-1}		ε_{33}^S	830.0 ε_0	
$\boldsymbol{s_{13}^E}$	-7.22×10^{-12} m^2 N^{-1}		ε_{11}^S	916.0 ε_0	
$\boldsymbol{\rho_p}$	7750.0 kg m^{-3}		ε_0	8.842×10^{-12} C mV^{-1}	

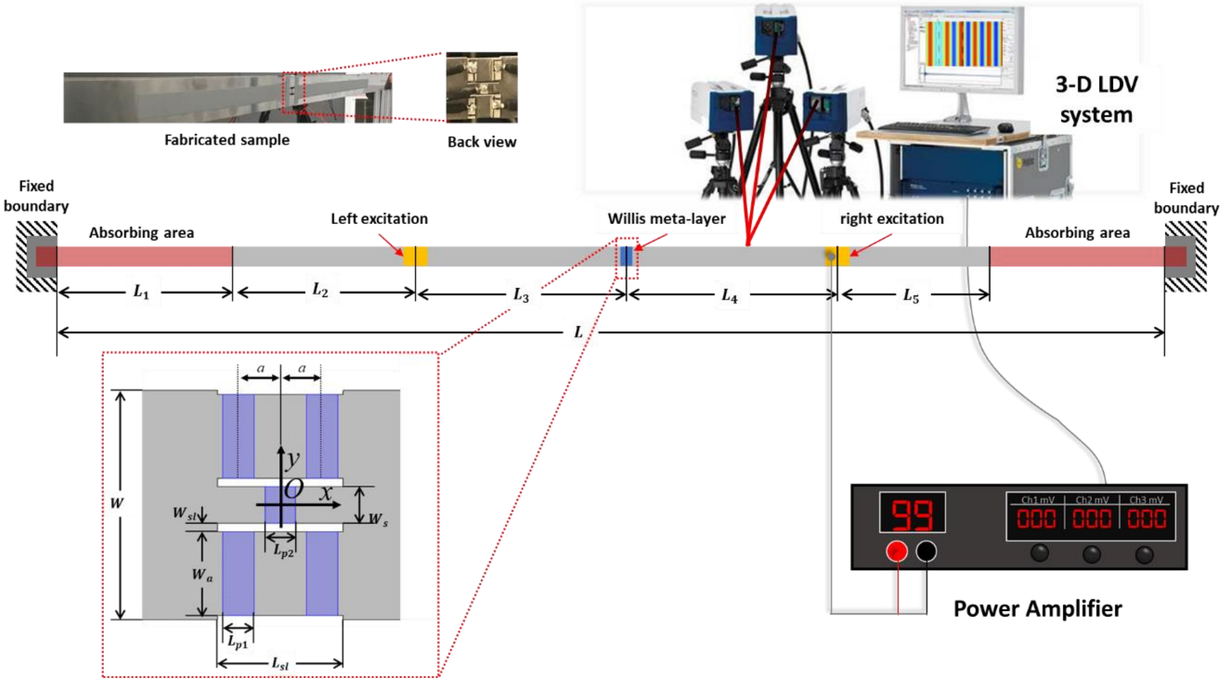

Supplementary Figure 4. Fabricated sample and experimental setup of the beam with Willis meta-layer.

Supplementary Note 6: Definition of the polarizability tensor for flexural waves in beams

We consider the mechanical active meta-layer as a point scatterer, and formulate its polarizability tensor, $\boldsymbol{\beta}$, for flexural waves as

$$\tilde{\mathbf{Q}} = \boldsymbol{\beta}\mathbf{F}_{\mathbf{loc}}, \tag{11}$$

where $\mathbf{F}_{\mathbf{loc}} = \begin{bmatrix} \psi_{loc} & w_{loc} & F_{loc} & M_{loc} \end{bmatrix}^{\mathrm{T}}$ is the incident wave field vector at the scatterer location, with ψ_{loc}, w_{loc}, F_{loc} and M_{loc} denoting local rotational angle, transverse displacement, shear force and bending moment, respectively. The vector $\tilde{\mathbf{Q}} = \begin{bmatrix} \tilde{q}_0 & \tilde{f}_0 & \tilde{s}_0 & \tilde{p}_0 \end{bmatrix}^{\mathrm{T}}$ is the multipole vector, representing the “excited” (scattered) field caused by the interaction of the point scatterer

with the local field, with $\tilde{q}_0$, $\tilde{f}_0$, $\tilde{s}_0$ and $\tilde{p}_0$ representing the body torque, transverse body force, shear strain and bending curvature, respectively. The definition provided in Eq. (11) describes general scattering behavior of flexural waves incident on an arbitrary point scatterer, which is analogous to definitions of electromagnetic and acoustic polarizability tensors. The physical representations of the multipole vector and associated radiation patterns in a host beam are shown in **Supplementary Figure 5**. It can be seen that torque and shear strain generate antisymmetric modes, where outward propagating waves are out-of-phase when traveling in opposing directions, while transverse force and bending curvature generate symmetric modes, where outward propagating waves are in-phase when traveling in opposing directions.

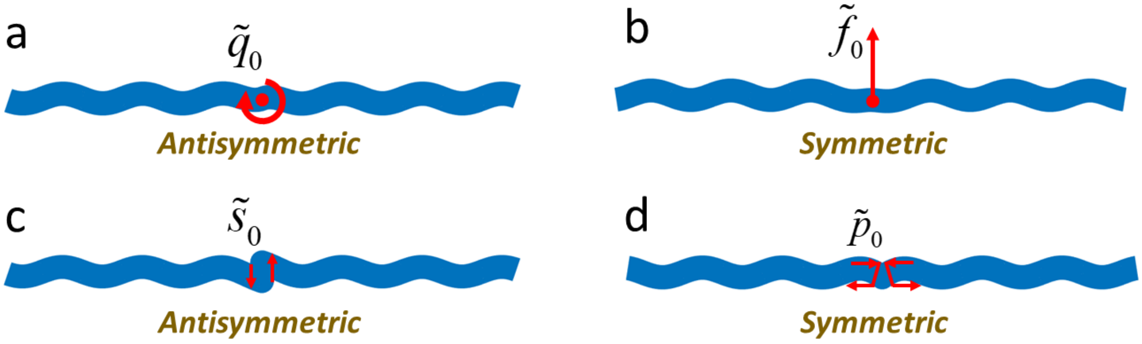

Supplementary Figure 5. Schematic of radiation patterns in the beam caused by the multipole vector $\tilde{\mathbf{Q}}$: (a) $\tilde{q}_0$; (b) $\tilde{f}_0$; (c) $\tilde{s}_0$; (d) $\tilde{p}_0$.

To determine orders of those multipole components of $\tilde{\mathbf{Q}} = \begin{bmatrix} \tilde{q}_0 & \tilde{f}_0 & \tilde{s}_0 & \tilde{p}_0 \end{bmatrix}^{\mathrm{T}}$, we conduct numerical simulations on a plate with excitations of body torque, body transverse force, shear strain and bending curvature applied in the middle portion of this plate. The out-of-plane displacement fields are shown in **Supplementary Figure 6**.

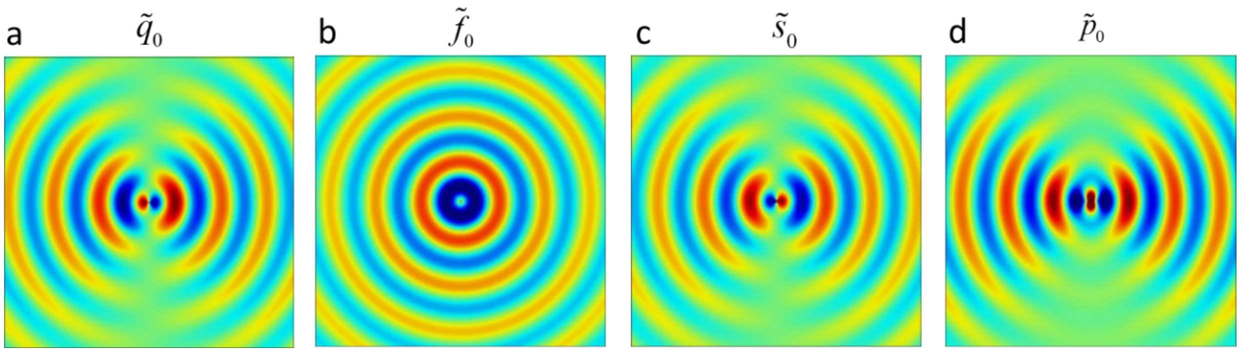

Supplementary Figure 6. Simulated out-of-plane displacement field on a plate with excitations of body torque (a), body transverse force (b), shear strain (c) and bending curvature (d).

As shown in **Supplementary Figure 6**, it is clear that $\tilde{f}_0$ is a monopole quantity of order zero in the multipole expansion and that $\tilde{q}_0$ and $\tilde{s}_0$ are dipole quantities of order one. Similarly, ψ_{loc}, w_{loc} and F_{loc} are dipole, monopole and dipole quantities, respectively. Furthermore, as shown in **Supplementary Figure 6c**, the field associated with localized curvature, $\tilde{p}_0$, results in a longitudinal quadrupole quantity. We thus justify $\tilde{p}_0$ and M_{loc} as longitudinal quadrupoles.

According to the definition of the polarizability tensor, its diagonal terms can be rewritten as $\beta_{11} = i\omega Z_{eff}^{(r)}$, $\beta_{22} = i\omega Z_{eff}^{(t)}$, $\beta_{33} = \frac{1}{G_{eff}}$ and $\beta_{44} = \frac{1}{D_{eff}}$, where $Z_{eff}^{(r)}$, $Z_{eff}^{(t)}$, G_{eff} and D_{eff} denote the effective impedance of rotational motion, effective impedance of transverse motion, effective shear stiffness and effective bending stiffness, respectively. These are physical meanings for diagonal terms. On the other hand, the off-diagonal terms represent all possible cross-couplings between local fields and excited multipoles, i.e. β_{13} and β_{31} represent antisymmetric-antisymmetric coupling between two dipole quantities, β_{24} and β_{42} represent symmetric-

symmetric coupling between monopole and multipole quantities, and β_{12}, β_{21}, β_{23}, β_{32}, β_{34}, β_{43}, β_{14} and β_{41} represent symmetric-antisymmetric couplings.

Supplementary Note 7: Polarizability-retrieval method for the meta-layer in the beam

To obtain all the 16 effective polarizability coefficients of the meta-layer in the 4×4 matrix, we formulate the polarizability-retrieval method (with Timoshenko beam assumption) step by step by considering both propagated (far-field) and evanescent (near-field) wave solutions.

First, kinematic equations of the Timoshenko beam with external deformation sources can be written as:

$$\begin{aligned} &\frac{\partial \psi}{\partial x} = \kappa + p, \\ &\frac{\partial w}{\partial x} - \psi = \gamma + s, \end{aligned} \tag{12}$$

where w, ψ, κ and γ respectively denote the transverse displacement, rotational angle, passive bending curvature and passive shear strain, while p and s respectively represent externally applied bending curvature and shear strain.

Second, conservation of the translational and rotational momentums of the Timoshenko beam with external loadings leads to the equations:

$$\begin{aligned} &\rho_0 \frac{\partial^2 w}{\partial t^2} - \frac{\partial F}{\partial x} = f, \\ &J_0 \frac{\partial^2 \psi}{\partial t^2} + \frac{\partial M}{\partial x} - F = q, \end{aligned} \tag{13}$$

where M, F, f, q, ρ_0 and J_0 respectively denote the bending moment, shear force, externally applied transverse body force and body torque, mass density, and rotational inertia per unit length of the host beam.

Thirdly, the constitutive relations of this host beam are expressed as:

$$\begin{aligned} M &= -D_0\kappa, \\ F &= AG\chi\gamma = G_0\gamma, \end{aligned} \tag{14}$$

where D_0, G, A and χ respectively denote the bending stiffness, shear modulus, area of the cross section and Timoshenko coefficient.

Combining Eqs. (12) - (14), the governing equation of the Timoshenko beam with external force/torque loadings and deformation sources can be obtained as

$$\begin{aligned} & D_0\frac{\partial^4 w}{\partial x^4} + \left(\frac{\rho_0\omega^2 D_0}{G_0} + J_0\omega^2\right)\frac{\partial^2 w}{\partial x^2} + \frac{\left(J_0\omega^2 - G_0\right)\rho_0\omega^2}{G_0}w \\ & = \left(-\frac{J_0\omega^2}{G_0} + 1\right)f - \frac{D_0}{G_0}\frac{\partial^2 f}{\partial x^2} - \frac{\partial q}{\partial x} + D_0\frac{\partial^2 p}{\partial x^2} + J_0\omega^2\frac{\partial s}{\partial x} + D_0\frac{\partial^3 s}{\partial x^3}, \end{aligned} \tag{15}$$

where the time harmonic term, $e^{i\omega t}$, is dropped from Eq. (15).

Considering a scatterer located at x = 0 in the Timoshenko beam. Its scattered waves due to the inhomogeneity can be equivalently regarded as "excited" waves caused by a multipole vector, $\tilde{\mathbf{Q}}$, with external force/torque loadings and deformation sources in the Timoshenko beam being $f = \tilde{f}_0\delta(x)$, $q = \tilde{q}_0\delta(x)$, $p = \tilde{p}_0\delta(x)$ and $s = \tilde{s}_0\delta(x)$. Those excited wave fields can be expressed in terms of Green's functions as:

$$w_s = \tilde{f}_0 G_f(x,0) + \tilde{q}_0 G_q(x,0) + \tilde{p}_0 G_p(x,0) + \tilde{s}_0 G_s(x,0), \tag{16}$$

where

$$\begin{aligned} G_f(x,0) &= A_1 e^{-ik_1|x|} + a_1 A_1 e^{-k_2|x|}, \\ G_q(x,0) &= \operatorname{sgn}(x)\left(A_2 e^{-ik_1|x|} + a_2 A_2 e^{-k_2|x|}\right), \\ G_p(x,0) &= A_3 e^{-ik_1|x|} + a_3 A_3 e^{-k_2|x|}, \\ G_s(x,0) &= \operatorname{sgn}(x)\left(A_4 e^{-ik_1|x|} + a_4 A_4 e^{-k_2|x|}\right), \end{aligned}$$

with

$$k_1 = -i\sqrt{\frac{-\alpha-\sqrt{\alpha^2-4D_0\beta}}{2D_0}} \quad , \quad k_2 = \sqrt{\frac{-\alpha+\sqrt{\alpha^2-4D_0\beta}}{2D_0}} \quad , \quad \alpha = \frac{\rho_0\omega^2 D_0}{G_0} + J_0\omega^2 \quad ,$$

$$\beta = \frac{\left(J_0\omega^2 - G_0\right)\rho_0\omega^2}{G_0}, \; A_1 = \frac{1}{2\left(ik_1 + a_1k_2\right)G_0}, \; a_1 = -\frac{\left(-\rho_0\omega^2 + G_0k_1^2\right)k_2}{\left(\rho_0\omega^2 + G_0k_2^2\right)k_1}i, \; A_2 = \frac{1}{2\left(ik_1 + c_0k_2\right)D_0b_0},$$

$$c_0 = \frac{i\left(\rho_0\omega^2 + G_0k_2^2\right)k_1}{\left(-\rho_0\omega^2 + G_0k_1^2\right)k_2} \quad , \quad b_0 = \frac{\left(-\rho_0\omega^2 + G_0k_1^2\right)}{iG_0k_1} \quad , \quad a_2 = -1 \quad , \quad A_3 = \frac{1}{2\left(b_0 - b_3\dfrac{b_0 + ik_1}{b_3 + k_2}\right)} \quad ,$$

$$b_3 = \frac{-\rho_0\omega^2 - G_0k_2^2}{G_0k_2}, \; a_3 = -\frac{b_0 + ik_1}{b_3 + k_2}, \; A_4 = \frac{1}{2\left(1 + d_0\right)}, \; d_0 = \frac{-\rho_0\omega^2 + G_0k_1^2}{\rho_0\omega^2 + G_0k_2^2} \text{ and } a_4 = 1.$$

The Green's functions satisfy the following:

$$\begin{aligned}
&D_0\frac{\partial^4 G_f(x,0)}{\partial x^4} + \left(\frac{\rho_0\omega^2 D_0}{G_0} + J_0\omega^2\right)\frac{\partial^2 G_f(x,0)}{\partial x^2} + \frac{\left(J_0\omega^2 - G_0\right)\rho_0\omega^2}{G_0}G_f(x,0) \\
&\qquad = \left(-\frac{J_0\omega^2}{G_0} + 1\right)\tilde{f}_0\delta(x) - \frac{D_0}{G_0}\frac{\partial^2\left[\tilde{f}_0\delta(x)\right]}{\partial x^2}, \\
&D_0\frac{\partial^4 G_q(x,0)}{\partial x^4} + \left(\frac{\rho_0\omega^2 D_0}{G_0} + J_0\omega^2\right)\frac{\partial^2 G_q(x,0)}{\partial x^2} + \frac{\left(J_0\omega^2 - G_0\right)\rho_0\omega^2}{G_0}G_q(x,0) \\
&\qquad = -\frac{\partial\left[\tilde{q}_0\delta(x)\right]}{\partial x}, \\
&D_0\frac{\partial^4 G_p(x,0)}{\partial x^4} + \left(\frac{\rho_0\omega^2 D_0}{G_0} + J_0\omega^2\right)\frac{\partial^2 G_p(x,0)}{\partial x^2} + \frac{\left(J_0\omega^2 - G_0\right)\rho_0\omega^2}{G_0}G_p(x,0) \\
&\qquad = D_0\frac{\partial^2\left[\tilde{p}_0\delta(x)\right]}{\partial x^2}, \\
&D_0\frac{\partial^4 G_s(x,0)}{\partial x^4} + \left(\frac{\rho_0\omega^2 D_0}{G_0} + J_0\omega^2\right)\frac{\partial^2 G_s(x,0)}{\partial x^2} + \frac{\left(J_0\omega^2 - G_0\right)\rho_0\omega^2}{G_0}G_s(x,0) \\
&\qquad = J_0\omega^2\frac{\partial\left[\tilde{s}_0\delta(x)\right]}{\partial x} + D_0\frac{\partial^3\left[\tilde{s}_0\delta(x)\right]}{\partial x^3}.
\end{aligned} \tag{17}$$

On the other hand, when the active meta-layer is excited with an incident wave, the scattered wave fields from the meta-layer (considered as a scatterer) can also be numerically extracted in the form of (see **Supplementary Figure 7**)

$$\begin{aligned} w_s^+ &= B_1 e^{-ik_1 x} + B_2 e^{-k_2 x}, \\ w_s^- &= B_3 e^{ik_1 x} + B_4 e^{k_2 x}, \end{aligned} \tag{18}$$

where B_1 and B_3 denote wave amplitudes of left- and right-propagating (far-field) waves, and B_2 and B_4 represent wave amplitude of left- and right- evanescent (near field) waves.

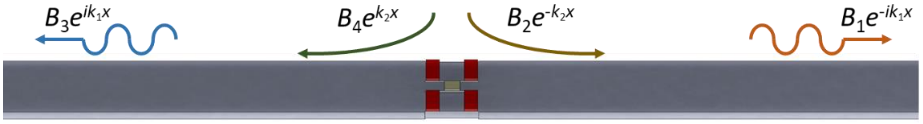

Supplementary Figure 7. Illustrations of coefficients of scattered wave fields.

Therefore, the excited multipole vector, $\tilde{\mathbf{Q}}$, of the meta-layer (scatterer) can be numerically determined by equalizing the excited wave fields in Eq. (16) and the scattered wave fields in Eq. (18) as

$$\begin{bmatrix} \tilde{f}_0 \\ \tilde{q}_0 \\ \tilde{p}_0 \\ \tilde{s}_0 \end{bmatrix} = \begin{bmatrix} A_1 & A_2 & A_3 & A_4 \\ A_1 a_1 & A_2 a_2 & A_3 a_3 & A_4 a_4 \\ A_1 & -A_2 & A_3 & -A_4 \\ A_1 a_1 & -A_2 a_2 & A_3 a_3 & -A_4 a_4 \end{bmatrix}^{-1} \begin{bmatrix} B_1 \\ B_2 \\ B_3 \\ B_4 \end{bmatrix}. \tag{19}$$

To retrieve the effective 4×4 polarizability tensor of the meta-layer attached with piezoelectric patches, four independent numerical tests are conducted. In the first two numerical tests (j = 1, 2), the transverse force source is located in the left or right ends of the host beam such that only propagating (far-field) waves can be measured by the active meta-layer (see Cases I and II in **Supplementary Figure 8**). In the last two numerical tests (j = 3, 4), the transverse force source is located adjacent to the left and right sides of the meta-layer such that both propagating (far-field)

and evanescent (near-field) waves can be measured by the active meta-layer (see Cases III and IV in **Supplementary Figure 8**).

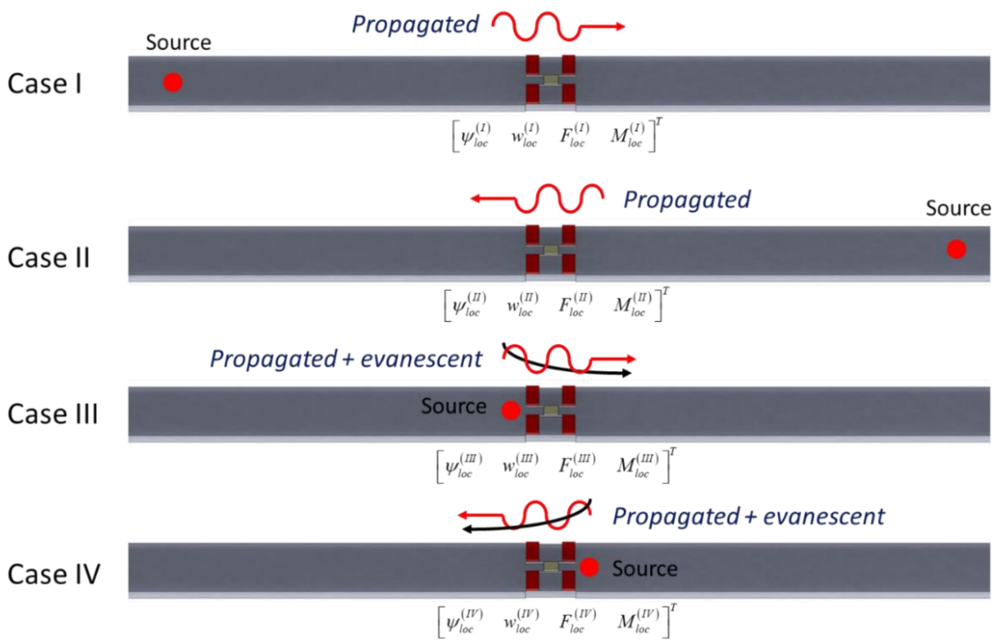

Supplementary Figure 8. Illustration of numerical tests under the four transverse forces at different locations.

For the j-th test, the excited multipole vector of the active meta-layer, $\tilde{\mathbf{Q}}^{(j)} = \begin{bmatrix} \tilde{q}_0^{(j)} & \tilde{f}_0^{(j)} & \tilde{s}_0^{(j)} & \tilde{p}_0^{(j)} \end{bmatrix}^{\mathrm{T}}$, is firstly determined based on Eq. (19), when the control is on. The local field vector at the active meta-layer location, $\mathbf{F}_{\mathbf{loc}}^{(j)} = \begin{bmatrix} \psi_{loc}^{(j)} & w_{loc}^{(j)} & F_{loc}^{(j)} & M_{loc}^{(j)} \end{bmatrix}^{\mathrm{T}}$, is then measured, when the control is off. Combining excited multipole vectors with their corresponding local field vectors for the four cases (j = 1, 2, 3, 4), one can retrieve the 16 polarizability coefficients, according to Eq. (11), as

$$\begin{bmatrix} \boldsymbol{\beta}_1 \\ \boldsymbol{\beta}_2 \\ \boldsymbol{\beta}_3 \\ \boldsymbol{\beta}_4 \end{bmatrix} = \begin{bmatrix} \mathbf{T}_{\mathbf{loc}}^{(11)} & \mathbf{T}_{\mathbf{loc}}^{(12)} & \mathbf{T}_{\mathbf{loc}}^{(13)} & \mathbf{T}_{\mathbf{loc}}^{(14)} \\ \mathbf{T}_{\mathbf{loc}}^{(21)} & \mathbf{T}_{\mathbf{loc}}^{(22)} & \mathbf{T}_{\mathbf{loc}}^{(23)} & \mathbf{T}_{\mathbf{loc}}^{(24)} \\ \mathbf{T}_{\mathbf{loc}}^{(31)} & \mathbf{T}_{\mathbf{loc}}^{(32)} & \mathbf{T}_{\mathbf{loc}}^{(33)} & \mathbf{T}_{\mathbf{loc}}^{(34)} \\ \mathbf{T}_{\mathbf{loc}}^{(41)} & \mathbf{T}_{\mathbf{loc}}^{(42)} & \mathbf{T}_{\mathbf{loc}}^{(43)} & \mathbf{T}_{\mathbf{loc}}^{(44)} \end{bmatrix}^{-1} \begin{bmatrix} \tilde{\mathbf{Q}}^{(1)} \\ \tilde{\mathbf{Q}}^{(2)} \\ \tilde{\mathbf{Q}}^{(3)} \\ \tilde{\mathbf{Q}}^{(4)} \end{bmatrix}, \tag{20}$$

where

$$\boldsymbol{\beta}_i = \begin{bmatrix} \beta_{i1} & \beta_{i2} & \beta_{i3} & \beta_{i4} \end{bmatrix}^{\mathrm{T}},\ i = 1, 2, 3, 4$$

$$\mathbf{T}_{\mathbf{loc}}^{(i1)} = \begin{bmatrix} \left(\mathbf{F}_{\mathbf{loc}}^{(i)}\right)^T \\ \mathbf{0} \\ \mathbf{0} \\ \mathbf{0} \end{bmatrix},\ \mathbf{T}_{\mathbf{loc}}^{(i2)} = \begin{bmatrix} \mathbf{0} \\ \left(\mathbf{F}_{\mathbf{loc}}^{(i)}\right)^T \\ \mathbf{0} \\ \mathbf{0} \end{bmatrix},\ \mathbf{T}_{\mathbf{loc}}^{(i3)} = \begin{bmatrix} \mathbf{0} \\ \mathbf{0} \\ \left(\mathbf{F}_{\mathbf{loc}}^{(i)}\right)^T \\ \mathbf{0} \end{bmatrix},\ \mathbf{T}_{\mathbf{loc}}^{(i4)} = \begin{bmatrix} \mathbf{0} \\ \mathbf{0} \\ \mathbf{0} \\ \left(\mathbf{F}_{\mathbf{loc}}^{(i)}\right)^T \end{bmatrix},\ i = 1, 2, 3, 4$$

To ensure that all terms in the tensor have the same units, we normalize polarizability tensors by firstly normalizing the left- and right-hand sides of Eq. (11) to the strain and then multiplying k_1 to the whole tensor as shown:

$$\begin{bmatrix} \beta'_{11} & \beta'_{12} & \beta'_{13} & \beta'_{14} \\ \beta'_{21} & \beta'_{22} & \beta'_{23} & \beta'_{24} \\ \beta'_{31} & \beta'_{32} & \beta'_{33} & \beta'_{34} \\ \beta'_{41} & \beta'_{42} & \beta'_{43} & \beta'_{44} \end{bmatrix} = \begin{bmatrix} \beta_{11}/G_0 & k_1\beta_{12}/G_0 & k_1\beta_{13} & k_1^2 D_0\beta_{14}/G_0 \\ \beta_{21}/(k_1 G_0) & \beta_{22}/G_0 & \beta_{23} & k_1 D_0\beta_{24}/G_0 \\ \beta_{31} & k_1\beta_{32} & k_1\beta_{33}G_0 & k_1^2 D_0\beta_{34} \\ \beta_{41}/k_1 & \beta_{42} & \beta_{43}G_0 & k_1 D_0\beta_{44} \end{bmatrix}. \qquad (21)$$

Supplementary Note 8: More results of the Willis meta-layer in a plate

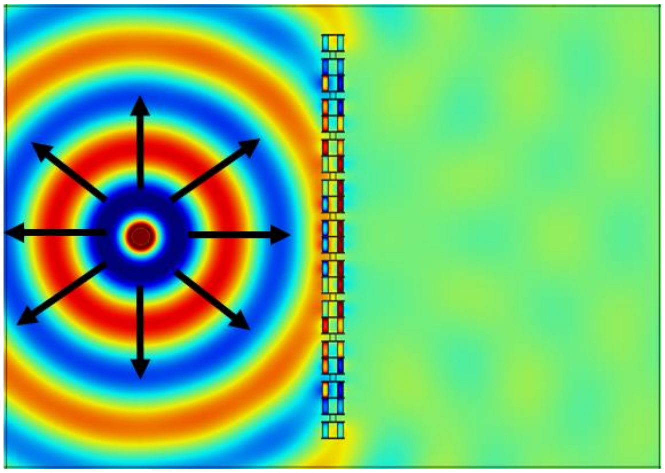

Supplementary Figure 9. Numerically simulated wave fields of total absorption with point incidence.

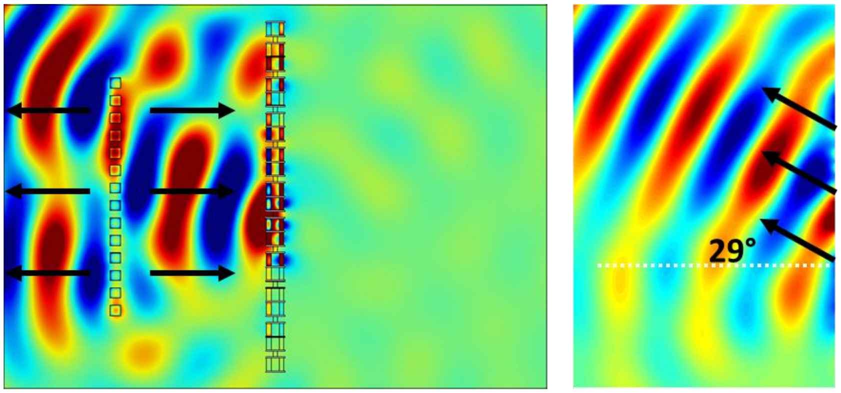

Supplementary Figure 10. Numerically simulated wave fields of wavefront transformations in the reflected domain.

Supplementary Note 9: Material parameters of Willis beams

Following homogenization approach in Ref [4], we suggest a general homogenization approach within the framework of Timoshenko beam theory by defining the general constitutive relation of Willis beams in a matrix form as

$$\begin{bmatrix} \kappa \\ J \\ \gamma \\ \mu \end{bmatrix} = \begin{bmatrix} -\bar{D} & S_{12} & S_{13} & S_{14} \\ S_{21} & \bar{I} & S_{23} & S_{24} \\ S_{31} & S_{32} & \bar{g} & S_{34} \\ S_{41} & S_{42} & S_{43} & \bar{\rho} \end{bmatrix} \begin{bmatrix} M \\ \dot{\psi} \\ F \\ V \end{bmatrix}, \tag{22}$$

where M, ψ, F and V denote the bending moment, rotational angle of the beam section, shear force, and transverse velocity, respectively, and κ, J, γ and μ represent the bending curvature, angular momentum, shear strain and linear momentum, respectively. In Eq. (22), $\bar{D}$, $\bar{I}$, $\bar{g}$ and $\bar{\rho}$ are the effective bending compliance, mass moment of inertia, inverse of the shear modulus, and mass density of the heterogeneous beam, respectively. The off-diagonal terms, S_{ij} ($i \neq j$), in the matrix denote the coupling coefficients of a Willis beam using Timoshenko theory. The Willis coupling in beams differs from that of 3D linear elastodynamic Willis constitutive form in that it exhibits

coupling between the higher order stress, bending curvature, local rotational angle and angular momentum.

Supplementary Note 10: Source-driven homogenization theory

We develop a source-driven homogenization theory to illustrate the connection between the polarizability tensor (Eq. (11)) and constitutive properties of Willis beams, which is a result of subwavelength periodic mechanical meta-layers (scatterers) embedded into a background beam.

Consider Timoshenko beam assumptions, conservations of translational and rotational momentums and kinematic equations of a beam are

$$\begin{aligned}\frac{\partial \psi}{\partial x} &= \kappa + p,\\ \frac{\partial V}{\partial x} &= \dot{\gamma} + \dot{\psi} + \dot{s},\\ \frac{\partial F}{\partial x} &= \dot{\mu} - f,\\ \frac{\partial M}{\partial x} &= -\dot{J} + F + q,\end{aligned} \tag{23}$$

where *q*, *f*, *s* and *p* are source terms that represent the externally applied body torque, transverse body force, shear strain and bending curvature, respectively. Assume source distribution terms with amplitudes p_{ext} , s_{ext} , f_{ext} and q_{ext} and time-harmonic term, $e^{i(kx+\omega t)}$, where k is the wavenumber along *x*-direction. Conservation relations in Eq. (23) for a background beam with those source distributions (see **Supplementary Figure 11a**) can be written in the spectrum domain as

$$\begin{aligned}ik\psi_{ext} &= -\bar{D}_0 M_{ext} + p_{ext},\\ ikW_{ext} &= \bar{g}_0 F_{ext} + \psi_{ext} + s_{ext},\\ ikF_{ext} &= -\omega^2 \bar{\rho}_0 W_{ext} - f_{ext},\\ ikM_{ext} &= \omega^2 \bar{I}_0 \psi_{ext} + F_{ext} + q_{ext}.\end{aligned} \tag{24}$$

where the mass density of the background beam is $\bar{\rho}_0$, bending compliance is $\bar{D}_0$, shear compliance is, $\bar{g}_0$, and rotational inertia is $\bar{I}_0$.

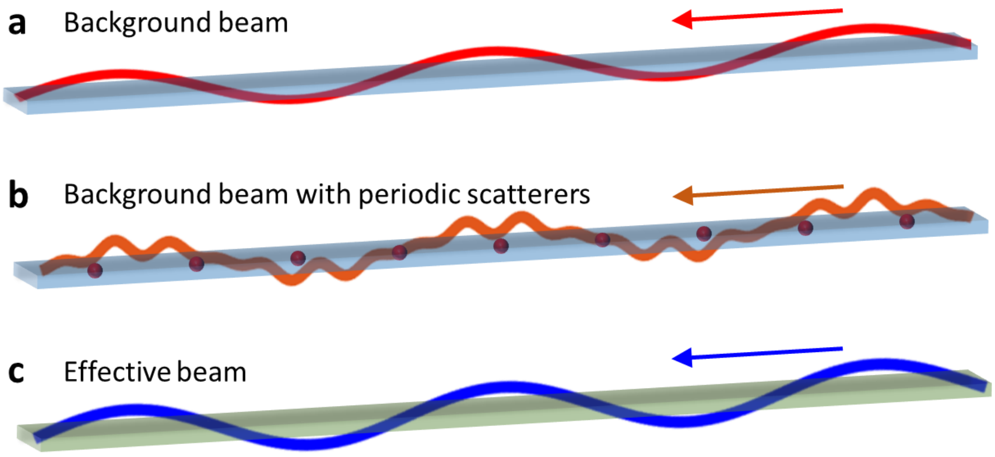

Supplementary Figure 11. Conceptual illustration of the source-driven homogenization procedure: (a) External sources applied on the background beam; (b) External sources applied on the background beam with periodic scatterers; (c) External sources applied on the effective beam.

We then introduce a periodic array of scatterers into the background beam (see **Supplementary Figure 11b**), conservations of transverse and angular momentums and the kinematic equations with the same source distributions in Eq. (28) read

$$\begin{aligned}
\frac{\partial \psi(x)}{\partial x} &= -\bar{D}_0 M(x) + \tilde{p}(x) + p_{ext} e^{ikx}, \\
\frac{\partial W(x)}{\partial x} &= \bar{g}_0 F(x) + \psi(x) + \tilde{s}(x) + s_{ext} e^{ikx}, \\
\frac{\partial F(x)}{\partial x} &= -\omega^2 \bar{\rho}_0 W(x) - \tilde{f}(x) - f_{ext} e^{ikx}, \\
\frac{\partial M(x)}{\partial x} &= \omega^2 \bar{I}_0 \psi(x) + F(x) + \tilde{q}(x) + q_{ext} e^{ikx},
\end{aligned} \tag{25}$$

where $\tilde{p}(x)=\left[-\bar{D}(x)+\bar{D}_0\right]M(x)$, $\tilde{s}(x)=\left[\bar{g}(x)-\bar{g}_0\right]F(x)$, $\tilde{f}(x)=\left[\omega^2\bar{\rho}(x)-\omega^2\bar{\rho}_0\right]W(x)$ and $\tilde{q}(x)=\left[\omega^2\bar{I}(x)-\omega^2\bar{I}_0\right]\psi(x)$, accounting for the contrast in bending compliance, shear compliance, mass density and rotational inertia, respectively, between the background beam and the inhomogeneities. For the continuous source distributions, the effective field amplitudes for a representative volume element can be uniquely determined by [3]

$$\begin{aligned} ik\psi_{eff} &= -\bar{D}_0 M_{eff} + \tilde{p}_{eff} + p_{ext}, \\ ikW_{eff} &= \bar{g}_0 F_{eff} + \psi_{eff} + \tilde{s}_{eff} + s_{ext}, \\ ikF_{eff} &= -\omega^2\bar{\rho}_0 W_{eff} - \tilde{f}_{eff} - f_{ext}, \\ ikM_{eff} &= \omega^2\bar{I}_0\psi_{eff} + F_{eff} + \tilde{q}_{eff} + q_{ext}, \end{aligned} \tag{26}$$

where $\tilde{p}_{eff}$, $\tilde{q}_{eff}$, $\tilde{s}_{eff}$ and $\tilde{f}_{eff}$ are effective sources of the bending curvature, body torque, shear strain and transverse body force caused by the scatterers, respectively. For the effective medium (see **Supplementary Figure 11c**), conservations of translational and rotational momentums and kinematic equations in the spectrum domain are

$$\begin{aligned} ik\psi_{eff} &= \kappa_{eff} + p_{ext}, \\ ikW_{eff} &= \gamma_{eff} + \psi_{eff} + s_{ext}, \\ ikF_{ext} &= \dot{\mu}_{eff} - f_{ext}, \\ ikM_{eff} &= -\dot{J}_{eff} + F_{eff} + q_{ext}. \end{aligned} \tag{27}$$

Comparing Eqs. (26) and (27), effective translational and rotational momentums, effective curvature and effective shear strain can be written

$$\begin{aligned} \kappa_{eff} &= -\bar{D}_0 M_{eff} + \tilde{p}_{eff}, \\ J_{eff} &= i\omega\bar{I}_0\psi_{eff} - \frac{\tilde{q}_{eff}}{i\omega}, \\ \gamma_{eff} &= \bar{g}_0 F_{eff} + \tilde{s}_{eff}, \\ \mu_{eff} &= i\omega\bar{\rho}_0 W_{eff} - \tilde{f}_{eff}. \end{aligned} \tag{28}$$

The purpose next is to find analytical formulas of $\tilde{p}_{eff}$, $\tilde{q}_{eff}$, $\tilde{s}_{eff}$ and $\tilde{f}_{eff}$ in terms of M_{eff} , ψ_{eff} , F_{eff} and W_{eff} . Multiple scattering effects are considered for this purpose.

Based on the retrieved polarizability tensor, sources of the bending curvature and shear strain in the multipole vector of each of the scatterers are

$$\begin{aligned} \tilde{p}_0 &= \beta_{44} M_{loc}, \\ \tilde{s}_0 &= \beta_{34} M_{loc}. \end{aligned} \tag{29}$$

The scattered wave fields at $x = x_m$ due to the n-th scatterer ($x = x_n$) can be written

$$M_s\left(x_m\right) = G_{pM}^{mn}\tilde{p}_n + G_{sM}^{mn}\tilde{s}_n, \tag{30}$$

where $G_{pM}^{mn} = R_{Mw}G_p\left(x_m, x_n\right)$ and $G_{sM}^{mn} = R_{Mw}G_s\left(x_m, x_n\right)$ with R_{Mw} being the ratio of the bending moment and transverse displacement. Green's functions $G_p\left(x_m, x_n\right)$ and $G_s\left(x_m, x_n\right)$ are defined in the Eqs. (16) and (17).

As a result, the local wave fields at the zeroth scatterer can be superposed as

$$M_{loc} = M_{ext} + \sum_{n\neq 0}\left(G_{pM}^{0n}\tilde{p}_n + G_{sM}^{0n}\tilde{s}_n\right). \tag{31}$$

We rewrite Eq. (31) into the following compact form

$$M_{loc} = M_{ext} + C_{pM}\tilde{p}_0 + C_{sM}\tilde{s}_0, \tag{32}$$

where

$$\begin{aligned} C_{pM} &= \sum_{n\neq 0} e^{ikx_n} G_{pM}^{0n}, \\ C_{sM} &= \sum_{n\neq 0} e^{ikx_n} G_{sM}^{0n}, \end{aligned}$$

where C_{pM} and C_{sM} are known as symmetric and antisymmetric lattice sums, respectively. Sources of the effective bending curvature and shear strain can be related to microscopic responses using spatial averages: $\tilde{p}_{eff} = \dfrac{\tilde{p}_0}{L}$ and $\tilde{s}_{eff} = \dfrac{\tilde{s}_0}{L}$ with L being the lattice constant of the inhomogeneous

beam. Combining Eqs. (24), (26), (28), (29) and (32) gives the effective constative relations of the inhomogeneous beam

$$\begin{aligned}
\kappa_{eff} &= -\left(\bar{D}_0 - \frac{\beta_{44}\Gamma}{L\Pi}\right)M_{eff}, \\
J_{eff} &= \bar{I}_0\dot{\psi}_{eff}, \\
\gamma_{eff} &= \bar{g}_0 F_{eff} - \frac{\beta_{34}\Gamma}{L\Pi}M_{eff}, \\
\mu_{eff} &= \bar{\rho}_0 V_{eff},
\end{aligned} \tag{33}$$

where

$$\Gamma = \frac{-k^4 + \bar{D}_0\omega^2\bar{\rho}_0 + \bar{D}_0\bar{I}_0k^2\omega^2 + \bar{g}_0k^2\omega^2\bar{\rho}_0 - \bar{D}_0\bar{I}_0\bar{g}_0\omega^4\bar{\rho}_0}{\begin{aligned}&\frac{ik\beta_{dM}\omega^2\bar{\rho}_0}{L\Pi} + \frac{\beta_{mM}\omega^2\bar{\rho}_0}{L\Pi} + \bar{D}_0\omega^2\bar{\rho}_0 - k^4 + \bar{D}_0\bar{I}_0k^2\omega^2 \\ &+ \frac{\bar{I}_0\beta_{mM}k^2\omega^2}{L\Pi} + \bar{g}_0k^2\omega^2\bar{\rho}_0 + \bar{D}_0\bar{I}_0\bar{g}_0\omega^4\bar{\rho}_0 + \frac{\bar{I}_0\beta_{mM}\bar{g}_0\omega^4\bar{\rho}_0}{L\Pi}\end{aligned}},$$

$$\Pi = 1 - C_{Md}\beta_{dM} - C_{Mm}\beta_{mM}.$$

The third equation in Eq. (33) displays the Willis coupling of the homogenized Willis beam, where the bending moment (symmetric) and shear strain (antisymmetric) are coupled. Comparing with Eq. (22), the effective material parameters of the Willis beam can be identified by matching terms:

$$\begin{aligned}
\bar{D} &= \bar{D}_0 - \frac{\beta_{44}\Gamma}{L\Pi}, \\
\bar{I} &= \bar{I}_0, \\
\bar{g} &= \bar{g}_0, \\
\bar{\rho} &= \bar{\rho}_0, \\
S_{31} &= -\frac{\beta_{34}\Gamma}{L\Pi}, \\
S_{ij} &= 0, \text{ for others.}
\end{aligned} \tag{34}$$

Equation (34) shows the analytical form of the connection between the polarizability tensor of the active scatterers considered in this work and the resulting effective constitutive properties of Willis beams using Timoshenko theory. From Eq. (34), it can be clearly seen that the effective mass

density, rotational inertia and shear compliance are left unchanged, and only one Willis coupling coefficient is nonzero. Applying conservation of translational and rotational momentum ($\dot{\mu} = \frac{\partial F}{\partial x} + f$, $\dot{J} = \frac{\partial M}{\partial x} - F + m$), we find that the coupling coefficient, S_{31}, will induce nonreciprocal wave propagation in the periodic Willis beam considered in this work. On the other hand, the effective bending stiffness of the Willis beam is modified by the polarizability, β_{44}, which is reciprocal.

Supplementary References

[1] Moheimani, SO Reza, and Andrew J. Fleming. Piezoelectric transducers for vibration control and damping. Springer Science & Business Media, 2006.

[2] Chen, Y., Li, X., Nassar, H., Hu, G. & Huang, G. A programmable metasurface for real time control of broadband elastic rays. Smart Mater. Struct. 27, 115011 (2018).

[3] Sieck, C. F., Alù, A. & Haberman, M. R. Origins of Willis coupling and acoustic bianisotropy in acoustic metamaterials through source-driven homogenization. Phys. Rev. B 96, 104303 (2017).

[4] Pernas-Salomón, R. & Shmuel G. Dynamic homogenization of composite and locally resonant flexural systems, J Mech. Phys. Solids 119, 43–59 (2018).
